# Risk Perception and Anxiety Regarding Radiation after the 2011 Fukushima Nuclear Power Plant Accident: A Systematic Qualitative Review

**DOI:** 10.3390/ijerph14111306

**Published:** 2017-10-27

**Authors:** Yoshitake Takebayashi, Yuliya Lyamzina, Yuriko Suzuki, Michio Murakami

**Affiliations:** 1 Department of Health Risk Communication, Fukushima Medical University School of Medicine, Fukushima 960-1295, Japan; ytake2@fmu.ac.jp; 2 Radiation Medical Science Center for the Fukushima Health Management Survey, Fukushima Medical University, Fukushima 960-1295, Japan; lyamzina@fmu.ac.jp; 3 Department of Adult Mental Health, National Institute of Mental Health, National Center of Neurology and Psychiatry, Tokyo 187-8553, Japan; yrsuzuki@ncnp.go.jp

**Keywords:** Fukushima disaster, radiation risk perception, radiation anxiety, risk communication

## Abstract

The purpose of this study was to provide a review of the publications of the risk perceptions or anxiety regarding radiation among people living in Japan after the 2011 Fukushima nuclear power plant accident. Two database (MEDLINE and PsycINFO) and hand-searched the references in identified publications were searched. For each identified publication, the measurements and time related-change of risk perception and anxiety regarding radiation were summarized. Twenty-four publications were identified. Quantitative measures of risk perception or anxiety were roughly divided into two types: single-item Likert scales that measure anxiety about radiation; and theoretical, or model-based measures. Rates of Fukushima residents with radiation-related anxiety decreased from 2012 to 2015. Factors governing risk perception or radiation-related anxiety were summarized by demographics, disaster-related stressors, trusted information, and radiation-related variables. The effects of risk perception or anxiety regarding radiation were summarized as severe distress, intention to leave employment or not to return home, or other dimensions. This review provides summary of current findings on risk perception or anxiety regarding radiation in Japan after the accident. Further researches are needed about detailed statistical analysis for time-related change and causality among variables.

## 1. Introduction

The Fukushima Daiichi nuclear power plant (hereafter FDNPP; note that the formal name of the plant is the Fukushima Daiichi Nuclear Power Station) accident following the Great East Japan Earthquake of 11 March 2011 released radionuclides that caused both internal and external exposure [1]. Although the additional effective dose was limited, anxiety regarding radiation remains a significant social issue in Japan. In this situation, risk communication plays a crucial role in management of public health, and a great deal of risk communication activities have been conducted in Fukushima and other affected areas. To conduct effective risk communication, it is suggested that a risk communicator should consider how residents perceive or react to hazards [2].

It has been determined that the FDNPP accident caused public anxiety about a variety of hazards, especially nuclear power and nuclear accidents [3,4,5]. In a nation-wide survey, Nakayachi et al. [3] compared anxiety to various hazards before (2008) and after the accident (2012). They found that anxiety over the prospect of a nuclear accident leapt after the accident (ranked 19 in 2008 and ranked 2 in 2012 of 51 hazards). A survey of residents of the Kansai area conducted by the Japanese Institute of Nuclear Safety System (INSS) has assessed anxiety about a nuclear accident using a 4-point Likert scale (0 = none, 1 = a little, 2 = quite, 3 = very) since 1993. In a summary of this survey, Kitada [4] revealed that 38% of respondents had a high level of anxiety (above “quite”) before the accident (in 2010). At four months after the accident (July 2011), the ratio of high-level anxiety increased to 83%. The Periodical Public Opinion Survey on Nuclear Energy for inhabitants living in the Tokyo Metropolitan Area also revealed that the percentage of people anxious about a nuclear accident increased from 49.8% in January 2011 to 70.8% in January 2012 [5].

Anxiety about radionuclides can be explained by the characteristics of human perception of risk. Slovic [6] divided risk perception into two psychological dimensions, namely “dread risk” and “unknown risk”. Members of the public perceive radiation risk and nuclear power plant accidents as high dread risks. The health effects of radiation are in line with both dimensions: dread risk perception is consistent with the involuntary exposure, the experience of the explosion, and anxiety among many residents about the effects of radiation on themselves, their children, and future offspring [7]; and the unknown risk perception is consistent with people’s feeling that the health effects of low-dose exposure are still poorly understood scientifically.

In general, risk perceptions are important factors affecting psychological distress [8], as well as individual and social decision-making [9]. After the Fukushima accident, risk perceptions regarding radiation have affected mental health [7,10] and decisions in regard to whether to evacuate, when to return home, and what to eat [11,12]. Although empirical data have been published during the 6 years after the Fukushima accident, no systematic qualitative review of the literature regarding risk perception has yet been published. 

Here we present a systematic review of studies of radiation perception and anxiety to allow a better understanding of radiation exposure, psychological distress, and physical health effects after the disaster [10,13,14,15]. This review summarizes the literature regarding risk perception or anxiety about radiation after the FDNPP accident in Japan. The specific objectives are to identify:

1. measures used to assess risk perception and anxiety;
2. the change of risk perception and anxiety with time and their modifiability by intervention;
3. factors governing risk perception and anxiety; and,
4. effects of risk perception and anxiety on residents.

## 2. Materials and Methods

**Figure 1:**
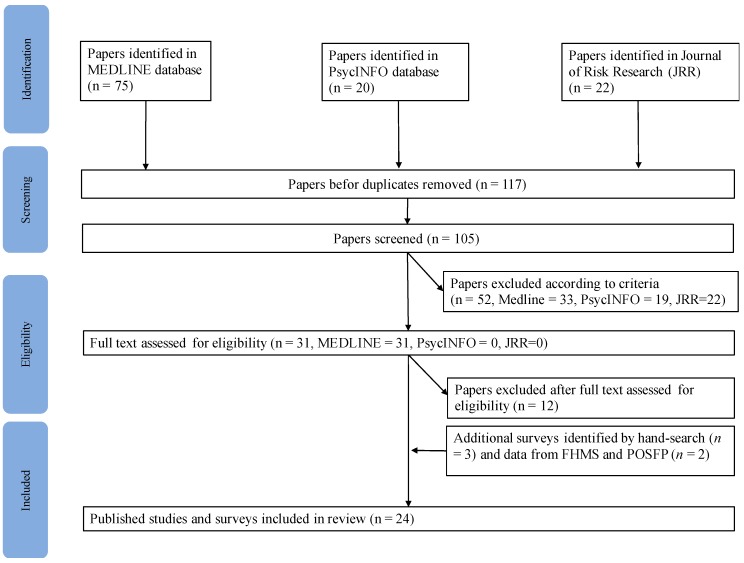
Flow diagram of study selection.

We searched the MEDLINE, PsycINFO online databases for publications published between March 2011 and 16 May 2017 and later hand-searched the references in those selected (Figure 1). We also searched the related field journal (Journal of Risk Research) not included in MEDLINE or PsycINFO.

Search keywords included “perception or anxiety or anxieties”, “radiation or radionuclide or radionuclides or radioactive”, and “Fukushima”, in different combinations. Two independent raters (YT and MM) screened the titles and abstracts of each article by the following criteria:

- Participants of the survey or intervention were living in Japan.
- Surveys included measurement of risk perception factors or of anxiety regarding radiation after the 2011 FDNPP accident.
- Factors governing risk perception and anxiety were investigated.
- The effects of risk perception and anxiety were investigated.

Included articles satisfied both criteria A and B, and either C or D. The consistency of raters’ evaluations was examined with Cohen’s kappa coefficient. Discrepancies between ratings were resolved via discussion between the raters. When considering that international readers’ accessibility and interpretation of original papers, we excluded articles written in languages other than English, chapters in books, and letters that did not include measurements of factors influencing radiation risk perception.

Initially, 117 studies were identified (Medline, 75; PsycINFO, 20; Journal of Risk Research, 22). After the duplicates were removed, 105 articles were screened. On the basis of the screening criteria and consensus discussion, 31 papers were retained. Inter-rater consistency was acceptable (κ = 0.72, *p* < 0.001). Evaluation of the full text against the eligibility criteria left 19 articles [7,11,12,16,17,18,19,20,21,22,23,24,25,26,27,28,29,30,31] in the final review. Another three articles [32,33,34] were found by hand-searching of the reference lists of the 19 articles. We also included published data from the Fukushima Health Management Survey (FHMS), conducted by Fukushima Medical University [35], and the Public Opinion Survey in Fukushima Prefecture (POSFP), conducted by the Fukushima Prefectural Government [36] from 2012 to 2015. Although these data do not meet the eligibility criteria, because either factors or effects were not investigated and POSFP is published in Japanese, we considered them to be useful in examining changes in risk perception or anxiety with time. The study characteristic and main findings are summarized in Supplementary Materials Tables S1–S3.

For each article, we summarized the measurements of risk perception and anxiety regarding radiation. Then, we summarized time-related changes. We divided articles into three categories: (a) factors governing risk perception or anxiety; (b) effects of risk perception or anxiety regarding radiation; (c) and, modifiability of risk perception and anxiety by intervention. These categories were based on the design or hypothesis of each article’s authors, and may not reflect actual causality.

## 3. Results

### 3.1. Characteristics of the Literature Included in This Review

The distribution of categories in the included papers is shown in Table 1. Twenty-two articles and the two surveys assessed risk perception or anxiety regarding radiation. Seven articles (29%) examined governing factors and explained the variance of risk perception [7,16,17,18,19,20,32], 10 (42%) examined the effects of risk perception or anxiety [7,11,12,21,22,23,24,25,33,34], and five (21%) [18,20,29,30,31] reported modifiability due to interventions (three of which also explain variance of risk perception factors). Three articles and the two surveys (20%) assessed risk perception and anxiety only [26,27,28,35,36], and did not examine governing factors and effects.

After final screening, we created four categories of subjects. General subjects included residents of Osaka, Tokyo, and Fukushima prefecture (both evacuated and not evacuated) and mothers living in Fukushima city, Miyagi prefecture, and Chiba prefecture (Tokatsu village). Employment-specific subjects included public health nurses, mental health nurses, general nurses, radiation decontamination workers, alpine workers, and rescue workers. Residents of evacuated areas came from Minamisoma city, and Kawachi village (both subject to evacuation orders). We also considered subjects who attended any explanatory meeting or risk communication workshop.

The study samples partly overlapped between Suzuki et al. [7] and Oe et al. [23], because they both used data from FHMS. Study samples also partly overlapped between Hino et al. [18] and Midorikawa et al. [29]. Other studies were conducted independently.

### 3.2. Measures of Risk Perception or Anxiety Regarding Radiation

Distribution of measurement type using in included papers is shown in Table 1. Quantitative measures of risk perception or anxiety were roughly divided into two types:

- single-item Likert scales that measure anxiety about radiation; and,
- theoretical or model-based measures.

Single-item Likert scales were the most frequently used measure (54%). They assessed overall or specific radiation-related anxiety. Overall, rating scales asked participants about their degree of radiation-related anxiety using a 1 (no anxiety) to 10 (having a lot of anxiety) or 1 (none) to 4 (very much) Likert scale. One study assessed respondents’ anxiety about radiation exposure using a binary response option (having anxiety: yes or no). Murakami et al. [22] asked about anxiety over radiation while also clarifying differences in anxiety over time (current radiation anxiety and change in radiation anxiety after the accident). In their study, current anxiety was assessed on a scale from 0 (very unworried) to 10 (very worried), while change in radiation anxiety after the accident was assessed on a scale with five choices (anxiety reduced compared to before, anxiety somewhat reduced compared to before, no change compared to before, anxiety somewhat increased compared to before, and anxiety increased compared to before). Sugimoto et al. [32] asked about the frequency of concerns about radiation (always, occasionally, indifferent, rarely, or never).

Specific anxiety related to the effects of radiation on the development of thyroid cancer [18,29] or on children or the workplace [12,26], estimation of the occurrence of acute radiation syndrome (an acute illness caused by irradiation of the entire body by a high dose of radiation in a short period of time) [24], or reluctance to eat foods grown in the evacuation order area [24].

Two theoretical models of risk perception were used: three studies used Slovic’s model [6] and four used Lindel’s model [37]. Slovic’s model considers dread risk and unknown risk [6]. Dread risk is typified by cancer risk, fatal effects on health, effects on future generations, and intuitive dread. Unknown risk relates to whether the health effects of radiation are not scientifically elucidated and whether the effects are not immediate [6,20]. Lindel’s model considers immediate health effects, delayed health effects, and genetic effects [37]. Two studies developed new scales to assess radiation risk perception and anxiety [19,31]. Some factors included in these scales correspond to Slovic’s or Lindel’s measures. Although Imamura et al. [31] and Sugimoto et al. [19] used new scales that were independently developed, common new, and unique factors were identified. These included effects of news reports on the accident and fears about social disruption. 

Yoshii et al. [26] asked women who were or became mothers around the time of the accident to describe their anxieties over radioactivity through the use of open-ended questions. They summarized responses through qualitative analysis and identified seven main concerns: food safety, outdoor safety, effects of radiation on gestating embryos, effects on the health of children, general radiation effects, economic factors (e.g., farmers’ worries about growing or selling produce), and feelings of distrust (related to announcements about food safety or radiation values in the media).

### 3.3. Changes over Time

Although no surveys or studies statistically tested changes in risk perception or anxiety over time, two articles reported descriptive data. Ito et al. [28] reported the frequency of and changes over time in the content of mothers’ opinions obtained in the FHMS Pregnancy and Birth Survey from 2011 to 2013. Mothers’ concerns about radiation decreased over time (rate of opinion about radiation effect on fetus: 29.8% in 2011, 26.8% in 2012, 17.4% in 2013). Kozaki et al. [27] surveyed residents, doctors, and medical students inside and outside Fukushima from 2011 to 2013. The rate of low anxiety increased from 27 to 53% among residents inside Fukushima. In contrast, doctors outside Fukushima and medical students reported consistently low anxiety.

**Figure 2:**
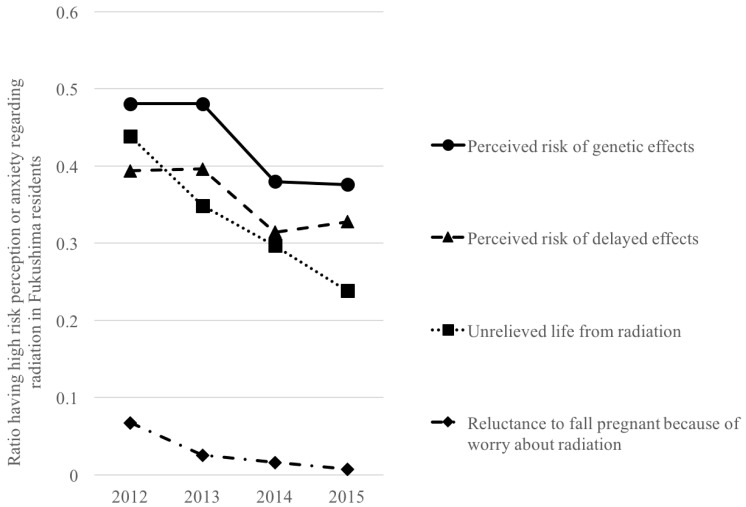
Change in ratios of high anxiety or risk perception regarding radiation over time in Fukushima residents [35,36].

Four indicators in FHMS and POSFP from 2012 to 2015 [35,36] reflect risk perception or anxiety: unwillingness to become pregnant because of worry about radiation, perceived risk of genetic effects, perceived risk of delayed effects, and unrelieved life from radiation. Overall, rates of Fukushima residents with radiation-related anxiety decreased from 2012 to 2015 (Figure 2).

### 3.4. Modifiability

Two descriptive and three statistical reports concerned modifiability of risk perception or anxiety by intervention. Fujii et al. [30] counseled mothers in Tokatsu village, Chiba prefecture, ~200 km from the FDNPP, where the ambient dose increased when compared with areas surrounding the village. After counseling, 78% of participants responded that communication consultation alleviated anxiety and fear of radiation exposure. Hino et al. [18] and Midorikawa et al. [29] reported that radiation-related anxiety was significantly reduced after an explanatory meeting about the effects of radiation on the thyroid gland; that anxiety tended to be reduced better if participants numbered <100; and that participants were satisfied with the objective data and cancer information provided. Sugimoto et al. [19] also reported a significant reduction in radiation-related anxiety among participants after radiation-health seminars conducted in 12 locations in Soma city, Fukushima prefecture.

Imamura et al. [31] used a randomized controlled trial to examine the effects of a mental health promotion program targeted at behaviors related to depressive mood, not to radiation-related anxiety. Mental health was improved but radiation-related anxiety remained unchanged.

### 3.5. Governing Factors

We summarized the factors governing risk perception or radiation-related anxiety by demographics [7,16,18,19,20,32], disaster-related stressors [7,20], trusted information [19,20], and radiation-related variables [16,17,18]. Generally, Slovic’s dread risk is more related to the degree of anxiety or to decision-making than unknown risk [6,20], so we focused on findings regarding dread risk.

#### 3.5.1. Demographics and Disaster Related-Stressors

Risk perception and anxiety were consistently high among women [7,18,19,20,32], the elderly [7,19], the married [20,32], parents of children [19,20], grandparents [19,20], the relatively poorly educated (having graduated only from middle or high school) [7,19], and those qualified in the humanities [20]. Risk perception and anxiety were also related to job [18,19,20] and habitation [7,18,19,32]. They were higher among people who were evacuated (both voluntarily and involuntarily), whose houses had been damaged, who experienced bereavement, who changed or lost jobs or homes, or who lost income [7,20].

#### 3.5.2. Trusted Information

Two studies examined the relationship between trusted information and risk perception or anxiety [19,20]. Murakami et al. [20] revealed that dread risk perception was greater among people who trusted direct information (from friends, online researchers, or others) than those who did not, but was lower among people who trusted central governmental information than among those who did not. Sugimoto et al. [19] examined the relationship between risk perception (using their original questionnaire) and frequency of use of mass media to obtain information about the disaster (rated as: “do not use”, “use once a week”, “use a few times a week”, and “use more than once a day”). Frequent use of rumor to obtain disaster information was related to high fear of radiation or health harms, use of regional newspapers was related to strong fears for the future, and use of radio was related to fears about social disruption, but use of national newspapers was negatively related to fears for the future.

#### 3.5.3. Radiation-Related Variables

Among participants at explanatory meetings about radiation and the thyroid, anxiety about radiation was related to safety behaviors (such as seeking information about radiation [18], checking dose rates and staying out of high-dose areas [17], and possessing educational materials about radiation [16]). The association of subjective knowledge with risk perception or anxiety was inconsistent between studies [16,18]. Radiation decontamination workers who had no one to consult or no written contract revealed more radiation-related anxiety than people who have consultation or written contract [17].

### 3.6. Effects

We summarized the effects of risk perception or anxiety regarding radiation as severe distress, intention to leave employment or not to return home, or other dimensions.

#### 3.6.1. Severe Distress

Six studies revealed that perception of risk is related to wellbeing (severe distress or life satisfaction). Suzuki et al. [7] and Oe et al. [23] related risk perception by Lindel’s model to severe mental illness observed in the FHMS. In particular, both studies revealed that the perceptions of genetic effects of radiation were strongly related to severe distress. Goto et al. [34] also found that concerns about radiation were significantly associated with depressive symptoms among mothers in Fukushima in the FHMS.

Consistent with findings from general Fukushima residents, Nukui et al. [21] revealed that dread risk perception in nurses was partly related to high-risk mental health problem, although other factors, such as ability to cope with daily life and work-related stressors were more important. Among medical assistance workers deployed to the disaster area, concern about radiation exposure was significantly associated with higher scores of psychological distress, depression, and post-traumatic stress symptoms one month after 11 March 2011 [33].

Murakami et al. [22] reported that increased anxiety about radiation after the accident was negatively related to satisfaction with life.

#### 3.6.2. Intention to Leave or Not Return

Radiation-related anxiety was related to intention to leave employment or not to return home [11,12]. A positive relationship between risk perception and intention to leave employment was observed among alpine workers and nurses who were working at the Fukushima Medical University hospital at the time of the accident [12,25]. Also, a positive relationship between risk perception and intention not to return home was observed in a study of residents in the evacuation order area [11].

#### 3.6.3. Other Dimensions of Risk Perception

Changes in anxiety about radiation were related to perception of dread risk [22]. Risk perception (delayed effects, genetic effects) was related to the perception that acute radiation syndrome might develop among the general population [24].

**Figure 3:**
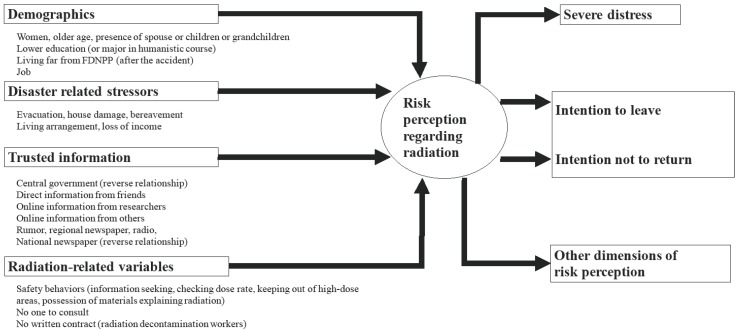
Contribution of risk factors to outcomes of risk perception or anxiety regarding radiation.

The effects and factors governing risk perception or anxiety are summarized in Figure 3.

## 4. Discussion

### 4.1. Measures

Single-item measures were frequently used to assess radiation-related anxiety. A simple measure is useful for assessing general or overall anxiety, and can also assess anxieties specific to subject characteristics. Theoretical or model-based measures were less frequently used. These allow generalizability or comparability because fixed items are used across various research contexts. It may be useful to include both measures to allow for both specificity and generalizability. For example, Murakami et al. [22] used both risk perception (dread and unknown) based on Slovic’s theory and single-item radiation-related anxiety, and how it changed after the accident.

There is a need to consider new scales or categories developed since the accident. If we rely only on traditional measures of risk perception, we may overlook important dimensions that are specific to the FDNPP accident and might apply to future nuclear accidents. Newly developed scales cover fear of social disruption and concerns about the effects of information from news reportage on the accident. Thus, it may be useful to assess perceptions about radiation itself, and about how to spread information related to radiation or social consequences, which is one of the most important features of effective risk communication following a nuclear accident.

### 4.2. Change over Time

Overall risk perception or anxiety tended to reduce over time. In particular, Lindel’s model of risk perception (genetic effects and delayed effects) showed a remarkable reduction from 2013 to 2014 in residents of Fukushima prefecture. Even following a nuclear accident, the human capacity for resilience might quickly reduce anxiety. One possible reason for this reduction is the implementation of various risk-countermeasure activities, including risk communication. However, the data may be biased because of participants’ characteristics (e.g., response rate among residents outside Fukushima; age and sex distribution) could not be consistent over time. Thus, changes in risk perception and anxiety with time and related factors should be examined statistically with properly adjusted covariates.

### 4.3. Modifiability

The findings of studies of explanatory meetings or one-to-one consultations with medical professionals indicate that such interventions may be useful in reducing radiation-related anxiety. This conclusion is consistent with risk communication situations that empirically shifted to face-to-face communications. The findings suggest that radiation-related anxiety can be modified through interventions. The studies also found that interventions may be useful for people with high radiation-related anxiety, and that communication is more effective among groups that have fewer than 100 participants. On the other hand, interventions that target factors other than radiation-related anxiety may not be useful, although it is possible that the effect of interventions targeted at mental health was overlooked owing to insufficient statistical power in Imamura et al. [31], which had a small sample size. This finding also implies that mental health can be improved without changes in risk perception or anxiety. Authorities or interveners should carefully consider the objectives of interventions. Risk perception or anxiety can work as a mediator of potential effects (e.g., severe distress, intention to leave or not return), but may not be a final outcome.

Following a nuclear accident, it may be unethical to conduct a randomized controlled trial to examine the effects of different interventions on anxiety. However, more sophisticated statistical analysis is needed to clarify the effect of interventions on the reduction of radiation-related anxiety or on the eventual outcome so as to help scientists or policymakers to better prepare for future accidents. The use of a counterfactual model of causal inference (e.g., propensity score method) could be used to examine causality.

### 4.4. Governing Factors and Effects of Risk Perception

The effects and factors governing risk perception or anxiety are summarized in Figure 3. Various variables were investigated as risk factors, but few outcomes were evident, notably severe distress and intention to leave or not return home.

Most factors governing risk perception, including sex, age, habitation, and presence of children generally agreed with findings in elements of environmental and social risk perception or communication [38,39,40]. In contrast, our finding of low education as a high risk perception factor contrasts with one study [39]. Importantly, newly found disaster-related factors included evacuation, house damage, and bereavement. This indicates that risk perceptions are governed not only by cultural worldviews established over a lifetime [41], but also experiences unique to the accident.

Although the importance of public communications that can the recognize perceived risks following disasters has been pointed out [42], the implementation of risk governance by governmental bodies or institutions is still very limited, despite the occurrence of 10 major chemical, radiological, and nuclear disasters since 1945 (e.g., Goiânia, Three Mile Island, Sellafield, Hiroshima and Nagasaki, Bhopal, Chernobyl). Yet all of these accidents have one main feature in common: experts regard the consequent mental health impact as the largest public health problem. Even 20 to 30 years later, stress-related symptoms and cognitive and psychological impairment remain high among affected populations. Stress-related disorders, such as depression and post-traumatic stress disorder, are significantly related to unaddressed risk perceptions, and are being diagnosed in Fukushima six years after the accident and were found in Chernobyl 11 years after the accident [43].

Furthermore, risk communication that ignores risk perception factors enhances mistrust among residents. High risk perception regarding radiation itself is natural; however, excessive reactions in avoiding radiation (e.g., loss of physical activity outdoors and good dietary habits) can cause risk tradeoff problems such as increased lifestyle diseases [44].

One way to resolve nuclear legacy problems is to develop a systematic methodology for socio-psychological support among affected groups living in territories affected by accidents or disasters that responds to cultural environment. Such an attitude will emphasize the importance of populations’ views, opinions, feelings, anxieties, and desires; in this way, risk perception factors related to contaminated environments will help governments and policymakers move toward more effective policymaking and management. For the ultimate success of risk communication, it is crucial to examine and compare the effects of risk perception among demographic groups and tailor risk messages to the concerns, fears, and anxieties of different groups among affected communities. Such an approach will help us improve nuclear safety by design, instead of in response to accidents [45].

### 4.5. Strengths and Weaknesses of This Study

This review refers to surveys done in Japan after the accident, and takes into account the location and the distance from the place of the accident. This study provides elements for grounded observations when different studies are matched. Also, the selection of articles and the four categories of findings were well defined. We identified methods for measuring risk perception or anxiety regarding radiation, together with the evolution in time of such anxiety and its effects. In conclusion, this review identified important new factors, and changes observed as a result of intervention. The elements collected by this review can be used to plan further studies and may be useful in developing a sound way to communicate risks.

However, this review also has some weaknesses. Most studies that were reviewed were cross-sectional surveys. The authors nominated the relational direction between variables (Figure 3), so any empirical relationships between variables are speculative. For example, it is unclear whether high risk perception causes severe distress or severe distress induces high risk perception. Further investigation is needed to clarify the relational direction. Cohort-type longitudinal multivariate data such as in FHMS may be suitable to examine this issue. In addition, detailed statistical investigations of changes in risk perception or anxiety over time are needed. Furthermore, there are no detailed studies examining the relationship between risk perception and individual doses of radiation exposure. Further studies are expected to reveal the relationship.

## 5. Conclusions

This review provides summary of current findings on risk perception or anxiety regarding radiation in Japan after the accident. Two types of methods were identified to measure risk perception or anxiety about radiation: single-item Likert scales and theoretical or model-based measures. Radiation-related anxiety decreased from 2012 to 2015 among Fukushima residents. Factors governing risk perception or radiation-related anxiety were summarized: demographics, disaster-related stressors, trusted information, and radiation-related variables. The effects of risk perception or anxiety regarding radiation included severe distress, intention to leave employment or not to return home, or other dimensions. Further researches are needed about detailed statistical analysis for time-related change and causality among variables.

## Figures and Tables

**Table 1:** Distribution of categories and measurement type used in the included papers.

Categories of Included Papers	Number of Papers	Ratio in All Included Papers (24)
Govering factors and explained the variance of risk perception	7	0.29
Effects of risk perception or anxiety	10	0.42
Modifiability due to intervention	5	0.21
Assessing risk perception and anxiety only	5	0.21
Measurement types		
Single item scale	13	0.54
Lindel’s model of risk perception	4	0.17
Slavic’s model of risk perception	3	0.13
Newly developed scale	2	0.08
Open-ended question or free opinion	2	0.08

## Supplementary Materials

The following are available online at www.mdpi.com/1660-4601/14/11/1306/s1, Table S1: Summaries of included articles examined governing factors of risk perceptions and anxieties regarding radiation after the FDNPP accident, Table S2: Summaries of included articles examined effect of risk perceptions and anxieties regarding radiation after the FDNPP accident, Table S3: Summaries of included articles examined time related change or modifiability of risk perceptions and anxieties regarding radiation after the FDNPP accident.
