# Supplementary material for: Risk Perception and Anxiety Regarding Radiation after the 2011 Fukushima Nuclear Power Plant Accident: A Systematic Qualitative Review
Source: Int J Environ Res Public Health. 2017 Oct 27;14(11):1306. doi: 10.3390/ijerph14111306 (PMC5707945; doi:10.3390/ijerph14111306)
Supplement: Supplementary file 1 [file ijerph-14-01306-s001.pdf]

International Journal of
Environmental Research and Public Health

Supplementary Materials: Risk Perception and Anxiety Regarding Radiation after the 2011 Fukushima Nuclear Power Plant Accident: A Systematic Qualitative Review

Yoshitake Takebayashi [1,2], Yuliya Lyamzina [2], Yuriko Suzuki [3] and Michio Murakami [1,2,*]

Table S1. Summaries of included articles examined governing factors of risk perceptions and anxieties regarding radiation after the FDNPP accident.

Citation	Sample Characteristics	N	Study Design	Period for Data Collection	Measure for Risk Perception or Anxiety	Factors Related to Risk Perception or Anxiety Regarding Radiation	Factors Related to Risk Perception or Anxiety Regarding Radiation	Adjusted Covariates
						Positive association	Negative association	
Suzuki et al. [7]	Residents of Fukushima	56 556	Cross sectional	Jan-12	**Lindell's risk perception model**<br>Immediate health effect	[Odds Ratio (99%CI) from multiple logistic regression]<br>**Demographics**<br>Age (>64 years) [1.78 (1.53–2.07)] (ref. 15–39 years),<br><br>**Disaster related stressors**<br>House damage (partial collapse and worse) [1.59 (1.39–1.82)]<br>Bereavement [1.46 (1.29–1.65)]<br>Living other than own house [1.22 (1.07–1.40)]<br>Decreased income[1.36 (1.19–1.55)]	[Odds Ratio (99%CI) from multiple logistic regression]<br>**Demographics**<br>Educational attainment: Vocational college, junior college or more [0.67 (0.58–0.77)] (ref. elementary, junior high or high school)	Sex, age, educational attainment, house damage, bereavement, living place, living arrangement at time of survey, type of work became unemployed, decreased income
					Delayed health effect	**Demographics**<br>Woman [1.20 (1.12–1.28)]<br><br>**Disaster related stressors**<br>House damage (partial collapse and worse) [1.28 (1.18–1.40)],<br>Bereavement [1.39 (1.29–1.50)],<br>Living out of Fukushima prefecture [1.19(1.10–1.29)],<br>Living other than own house [1.17 (1.08–1.26)],<br>Became unemployment [1.23(1.13–1.33)],<br>Decreased income[1.39 (1.29–1.50)]	**Demographics**<br>Educational attainment: Vocational college, junior college or more [0.83 (0.77–0.90)]<br>(ref. elementary, junior high or high school)	Sex, age, educational attainment, house damage, bereavement, living place, living arrangement at time of survey, type of work became unemployed, decreased income
					Genetic effect	**Demographics**<br>Age (>64 years) [1.78 (1.53–2.07)] (ref. 15–39 years),<br>Age (>50–64 years) [1.12 (1.04–1.20)]	**Demographics**<br>Educational attainment: Vocational college, junior college or more [0.81 (0.76–	Sex, age, educational attainment, house damage, bereavement, living place, living arrangement at time of survey, type of work became

						(ref. 15–39 years),<br>Woman [1.20 (1.12–1.28)]<br><br>**Disaster related stressors**<br>House damage (partial collapse and worse) [1.22 (1.12–1.32)]<br>Bereavement [1.42 (1.32–1.52)]<br>Living out of Fukushima prefecture [1.08(1.00–1.17)]<br>Living other than own house [1.14 (1.07–1.22)]<br>Became unemployment [1.26(1.17–1.36)]<br>Decreased income[1.39 (1.29–1.49)]	0.87)] (ref. elementary, junior high or high school)	unemployed, decreased income
Yoshida et al. [16]	Public health nurse in the Fukushima prefecture	430	Cross sectional	Jul-15	**Single item overall rating**<br>Single item 10 point likert scale for radiation anxiety (ranging from no anxiety (1) to having a lot of anxiety (10)<br>1 to 5 anxiety(-), 6 to 10 anxiety (+)	[Odds ratio (95%CI) from multiple logistic regression]<br>**Demographics**<br>Public health nurse at the time of the accident [2.37 (1.27 to 4.42)]<br><br>**Radiation related-variables**<br>Currently have materials to obtain knowledge about radiation [2.11 (1.25 to 3.60)]<br>Knowledge about childhood thyroid cancer increase after the Chernobyl accident [1.69 (1.04 to 2.75)]		Age, manager in the workplace, public health nurse at the time of the accidents, difficulty answering radiation questions in the past
Hidaka et al. [17]	Radiation decontamination workers in Fukushima prefecture	1505	Cross sectional	Oct-13	**Single item overall anxiety**<br>How much anxiety do you have over radiation exposure ?The answers were then measured on a four-point scale (1 = " Very much, " 2 = "Somewhat," 3="A little bit," and 4="None").	[Odds ratio (95%CI) from multiple logistic regression]<br>**Radiation related-variables**<br>Checking the dose rate and keeping out of high dose areas [0.375 (0.006, 0.744)]	[Odds ratio (95%CI) from multiple logistic regression]<br>**Radiation related-variables**<br>Written contract with current company regarding vacation, wages, and perquisite [-0.605 (-1.188, -0.021)],<br>Close persons for consultation [–0.454 (–0.873, -0.034)]	Age, previous residence, radiation passbook, Public assistance, training sessions, watching a video, physical condition check, self-study with materials, self-study without materials, monitoring external exposure, wearing a mask, wearing a radiation protection suit
Hino et al [18]	Explanatory meeting participants (749 people in Fukushima and 50 outside Fukushima (in Yamagata) participated)	749	Cross sectional	Sep-14 to June-15	**Single item specific anxiety**<br>Likert scale for anxiety regarding the effects of radiation on the thyroid (1–10) befor explanatory meeting	[Mann-Whitney U test, or Kruskal-Wallis test with Bonferroni correction]<br>**Demographics**<br>Sex: man < woman<br>Job: teaching staff, and municipal employee < guardians < test subjects<br>Region of meeting: Soso < Kenpoku,	[Mann-Whitney U test, or Kruskal-Wallis test with Bonferroni correction]	No adjustment

						kenchu < iwaki < Outside of Fukushima<br><br>**Radiation related variables**<br>Attitudes about radiation: collecting information about radiation, no one with whom to talk about radiation, no understanding about radiation	**Radiation related variables**<br>Attitudes about radiation: having family with whom to talk about radiation, having friends with whom to talk about radiation, not knowing about radiation	
Sugimoto et al. [19]	Residents in Soma city: Participants in a radiation-health-seminar for a total of 1560 residents, at 12 different locations in Fukushima	1560	Cross sectional	Jun-11 to Jul-11	**Original scale**<br>The questionnaire contained 14 items addressing fears about radiation and health, future social and economic prospects, and the current social and physical circumstances in which respondents lived.	[multiple linear standardized regression coefficient (95%CI)]	[multiple linear standardized regression coefficient (95%CI)]	Age, sex, job, total number of media types used, cancer history, living area, living with children or gland children, media type
					Radiation/health fear	**Demographics**<br>Age: under 35 [0.24 (0.05 to 0.43)], between 35 to 49 [0.22 (0.07,0.36)] (ref. over 50), Woman [0.30 (0.17, 0.44)], Area (seaside < mountain): 0.30 (0.12 to 0.48),<br>Living with grandchild (yes > no) 0.30 (0.12 to 0.49)<br><br>**Trusted information**<br>Use of rumors [0.22 (0.09 to 0.35)],		
					Fears for future	**Demographics**<br>Lower educational level [0.31 (0.13 to 0.49)],<br>Outdoor worker 0.59 (0.35 to 0.83), (ref. housewife),<br>Medical/education/technology worker [0.28 (0.02 to 0.39)] (ref. housewife),<br>Living with children [0.14 (0.01 to 0.27)]<br><br>**Trusted information**<br>Use of regional newspapers [0.18 (0.04, 0.32)]	**Trusted information**<br>Use of national newspapers[-0.14 (-0.26, -0.01)]	
					Fears about social disruption	**Demographics**<br>Elderly [20.18 (20.34 to 20.02)], Woman		

						[0.30 (0.19 to 0.42)]<br><br>**Trusted information**<br>Use of radio [0.16 (0.05,0.27)]		
Murakami et al. [20]	General population (from Tokyo, Osaka, Fukushima)	9249	Cross sectional	Dec-15	**Slovic's model of risk perception**<br>Dread risk	[Multiple linear regression coefficient (95%CI), standardized regression coefficient]<br>**Demographics**<br>Fukushima (evacuated) [0.131 (0.017, 0.246), 0.023] (ref. Osaka),<br>Woman [0.140 (0.100–0.180, 0.074],<br>Presence spouse [0.070 (0.020, 0.121), 0.037]<br>Presence children [0.122 (0.071, 0.172), 0.064]<br>Smoking habit [0.077 (0.029, 0.125), 0.033]<br><br>**Trusted information**<br>Trust for direct information from friend [0.161 (0.087, 0.236), 0.044]<br>Trust for online information from researcher [0.088 (0.036, 0.140), 0.037]<br>Trust for online information from others [0.210 (0.131, 0.288), 0.059]	[multiple linear regression coefficient (95%CI), standardized regression coefficient]<br>**Demographics**<br>Fukushima (not evacuated) [-0.290 (-0.347, -0.233), 0.102] (ref. Osaka) ,<br>Neither in science or humanities course [-0.078 (-0.130, -0.025), 0.031] (ref. humanities),<br>Major in science course [-0.078 (-0.130, -0.025), 0.031] (ref. humanities)<br><br>**Trusted information**<br>Trust for TV and radio [-0.051 (0.010, 0.092), 0.026],<br>Trust for central government [-0.251 (-0.303, -0.198), -0.100],	Habitation (Tokyo, Osaka, Fukushima (evacuated, not evacuated), sex, age, employee, spouse, children, grandchildren, educational status, smoking, trusted information (TV and radio, researcher-direct, newspapers, central government, friends-direct, researcher-online, others-online)
					Unknown risk	[multiple linear regression coefficient (95%CI), standardized regression coefficient]<br>**Demographics**<br>Woman [0.076 (0.038, 0.114), 0.044],<br>Presence children [0.111 (0.077–0.146) 0.065] (ref. absence of children etc.),<br>Smoking habit 0.080 [(0.036, 0.123), 0.038]<br><br>**Trusted information**<br>Trust for TV and radio [0.128 (0.091, 0.164), 0.073],<br>Trust for direct information from friend [0.135 (0.068, 0.203)], 0.041],<br>Trust for online information from others	[multiple linear regression coefficient (95%CI), standardized regression coefficient]<br>**Demographics**<br>Fukushima (not evacuated) [-0.261 (-0.312, -0.209), -0.102] (ref. Osaka),<br>Self-employment [-0.104 (-0.174, -0.034), -0.031] (ref. company employees etc.)<br><br>**Trusted information**<br>Trust for central government-[0.178 (-0.225, -0.130), -0.079],	Habitation (Tokyo, Osaka, Fukushima (evacuated, not evacuated), sex, age, employee, spouse, children, grandchildren, educational status, smoking, trusted information (TV and radio, researcher-direct, newspapers, central government, friends-direct, researcher-online, others-online)

						[0.109 (0.043, 0.176), 0.034]	Trust for direct information from researcher [-0.085 (-0.131, -0.038), -0.038]
Sugimoto et al [32]	National wide random subjects	5809	Cross sectional	Mar-12	**Single item overall rating** Concern about radiation	[Odds ratio (95%CI) from multiple logistic regression] **Demographics** Woman [1.67 (1.35–2.06)], Married [1.53(1.33–1.77)], Tohoku region invoking of disaster relief act [3.36(2.25–5.01)], Kanto region [2.08 (1.58–2.74)]	[Odds ratio (95%CI) from multiple logistic regression] **Demographics** Student [0.72(0.53–0.98)]

Table S2. Summaries of included articles examined effect of risk perceptions and anxieties regarding radiation after the FDNPP accident.

Effect of Anxiety about Radiation							
**Citation**	**Subject characteristics**	**N**	**Study design**	**Period for data collection**	**Measure for risk perception or anxiety**	**Outcome of risk perception or anxiety regarding radiation**	**Adjusted covariates**
Suzuki et al. [7]	Residents of Fukushima	56 556	Cross sectional	Jan-12	**Lindell's risk perception model**<br>Immediate health effect<br>Delayed health effect<br>Genetic effect	[Odds Ratio (95%CI) from multiple logistic regression]<br>Severe psychological distress [1.64 (1.42–1.89)]<br>Severe psychological distress [1.48 (1.32–1.67)]<br>Severe psychological distress [2.17 (1.94–2.42)]	Individual characteristics and disaster-related stressors
Orita et al. [11]	Kawauchi Municipal Government officer	127 (71: do not return, 56: had returned)	Cross sectional		**Single item overall radiation anxiety**<br>Anxiety to radiation exposure (yes or no)<br>Anxiety to radiation exposure by eating (yes or no)	[Odds Ratio (95%CI) from multiple logistic regression]<br>Anxiety to radiation exposure on intention do not return to hometown [8.91 (3.23–24.58)]	Sex, measured dose rate at their home, Employment, difficulty of being away from familiar stores, anxiety to radiation exposure by eating
Takeda et al. [12]	Alpine employees	343	Cross sectional	Oct-14 to Jun-15	**Single item specific radiation effect**<br>Did you feel considerable effects of radiation while at work?	[Odds Ratio (95%CI) from multiple logistic regression]<br>Greater risk perception about influence of radiation on the workplace predict intention to leave their jobs [0.33 (0.14–0.80)]	Age, born in Fukushima, marital status, living with preschool child, measurement of the ambient dose rate in the house after the accident, Anxiety about relationships with colleagues after the accident, A person to consult about radiation in the workplace
Nukui et al. [21]	Mental health hospital nurse in Fukushima prefecture	730	Cross sectional	Aug-15, Nov-15	**Slovic's model of risk perception**<br>Dread risk<br>Lindell's model of risk perception<br>Immediate effect<br>(effect on own health)<br>Unknown risk<br>Controllability	[Odds Ratio (95%CI) from multiple logistic regression]<br>High risk mental health [1.19 (1.05–1.34)]<br>High mental health [0.87 (0.78–0.98)]<br>not significant.<br>not significant.	
Murakami et al. [22]	Residents in Marumori Town, Igu County, Miyagi prefecture	698	Cross sectional	Mar-15	**Slovic's risk perception model**<br>Dread risk<br>Unknown	[Odds Ratio (95%CI) from multiple logistic regression]<br>Reduction radiation anxiety [OR: 0.22 (95%CI 0.11–0.45)]<br>not significant.	Sex, age, presence of a jobless person, subjective feeling on health, evaluation of announcements, evaluation of decontamination, evaluation of whole body counter
					**Single item overall rating**<br>Change anxiety about radiation after	[Odds Ratio (95%CI) from multiple logistic regression]	Sex, age, presence of a jobless person, subjective feeling on health, sense of attachment to the town, importance of

					the accident<br><br>Current anxiety about radiation	Satisfaction with life<br>for low current anxiety [3.09 (1.22–7.84)], for high current anxiety [3.00 (1.17–7.74)]	happiness
Oe et al. [23]	Residents who were registered in the municipalities categorized as complete evacuation order areas	12371	Cross sectional	Jan-13	**Lindell's risk perception model**<br>Delayed effect of radiation<br><br>Genetic effect of radiation	not significant.<br>[Odds Ratio (95%CI) from multiple logistic regression]<br>not significant.<br><br>Severe distress trajectory group [3.91 (3.17–4.83)]	Sex, age at disaster,<br>health-related variables<br>problem drinking, subjective sleep insufficiency, poor social support<br>disaster-related variables<br>disaster related home damage, disaster related bereavement, relocation 5 times or more after the disaster
Orita et al. [24]	Residents of Kawauchi village	285	Cross sectional	May-14 to Jun-14	**Lindell's risk perception model**<br><br>Delayed effect<br>(health effects on children)<br><br>Genetic effect<br>(health effects on offspring)	[Odds Ratio (95%CI) from multiple logistic regression]<br><br>Risk perception for acute radiation syndrome (ARS) might develop for general population by the FDNPP accident [31.01 (3.35, 286.65)]<br><br>Risk perception for acute radiation syndrome (ARS) might develop for general population by the FDNPP accident [4.73 (1.25, 17.90)]	Sex, age, Did you live in Kami-Kawauchi, not Shimo-Kawauchi before the accident?, Are you reluctant to eat rice or vegetables produced in the village?, Are you reluctant to radiological examination in the hospital?
					**Original items**<br>Do you have anxiety about health effects would appear in the general population simply by living in an environment with a 0.23 μSv per hour ambient dose for one year?<br><br>Reluctant to eat food produced in the village<br><br>Do you believe that adverse health effects would occur in the general population by eating 100 Bq per kg of mushrooms for one year?	Risk perception for acute radiation syndrome (ARS) might develop for general population by the FDNPP accident [6.87 (2.67, 17.71)]<br><br>not significant.<br><br>Risk perception for acute radiation syndrome (ARS) might develop for general population by the FDNPP accident [3.54 (1.13–11.12)]	Sex, age, Did you live in Kami-Kawauchi, not Shimo-Kawauchi before the accident?, Are you reluctant to eat rice or vegetables produced in the village?, Are you reluctant to radiological examination in the hospital?
Sato et al. [25]	Nurses who were working at the	345	Cross sectional	Jun-13	**Lindell's risk perception model**<br>Perception of adverse health effects	[Odds Ratio (95%CI) from multiple logistic regression]	Experience in nursing the patients with radiation therapy, Relatively high ambient dose rate around the house ,

	Fukushima Medical University Hospital at the time of the FDNPP					Subjects who were considered of the possible radiation health effects in children tend to have intention to leave their jobs than not considered [1.96 (1.02–3.54)]	Consideration of evacuation from Fukushima, living with preschooler , Anxiety about life in Fukushima city after the accident (no < yes), Anxiety about relationships with colleagues in the hospital after the accident (no < yes)
Matsuoka et al. [33]	Disaster medical assistance team workers deployed at the disaster area	424	Cross sectional	Apr-12	**Single item overall rating** Were you concerned over radiation exposure during the deployment? (yes or no)	[Analysis of covariance] Psychological Distress (K6), Depression (CES-D), Posttraumatic symptom (PDI or IES-R) were greater among workers who have concerned over radiation exposure than workers who have not.	Age, occupation, disaster operation experience, duration of time spent watching earthquake news, past history of psychiatric illness
Goto et al. [34]	Women who registered their pregnancies in Fukushima prefecture	6686 in 2012 6423 in 2013	Cross sectional	2012–2013	**Single item overall rating** Concern about radiation, defined as bottle feeding their babies because of radiation concerns (vs breast feeding, bottle feeding as a result of lack of breast milk, or bottle feeding for other reasons)	[Odds Ratio (95%CI) from multiple logistic regression] Concern about radiation were significantly associated with depressive symptoms. [3.41 (2.59–4.50)]	Age, postpartum days, at the time of survey, first-time motherhood, obstetrical complications

Table S3. Summaries of included articles examied time related change or modifiability of risk perceptions and anxieties regarding radiation after the FDNPP accident.

Citation	Subject Characteristics	N	Study Design	Period for Data Collection	Measure for Risk Perception or Anxiety	Time Related Change or Modifiability
Assessment only						
Yoshii et al. [26]	Study participants were mothers who had delivered a child less than one month before the Great East Japan Earthquake that occurred on March 11, 2011, and pregnant women	259	Cross-sectional descriptive	Jul-12	Item derived from open-ended question regarding radiation anxiety. anxiety about food safety, safety of the outdoors, effects of radiation on embryos during pregnancy, effects on children, radiation effects, Economy, distressful feelings about announcements	
Time related change						time related change
Kohzaki et al. [27]	1) Residents inside or 2) outside Fukushima, 3) Doctors inside or 4) outside Fukushima, and 5) medical student	1) 2013: 422, 2011: 959<br>2) 2013: 93, 2011: 31<br>3) 2013: 80, 2011: 83<br>4) 2013: 227, 2011: 388<br>5) 2013: 108, 2011: 106	Cross sectional cohort	Sep-11 to Nov-11<br><br>Aug-13 to Nov-13	How do you feel about the future radiation effect after FDNPP accident?<br>(a) No anxious, (b) Slightly anxious, (c) Anxious, (d) Very anxious, (e) Unknown	The response ratio low anxiety about radiation (including response no anxiety and slight anxiety) changed from 27 to 53% among residents inside Fukushima, no anxiety changed from 8 to 37% among residents outside Fukushima, no anxiety changed from 21 to 40% among doctors inside Fukushima overtime (from 2011 to 2013).
Ito et al. [28]	Women who received a Maternal and Child Health Handbook either from a municipal office in another prefecture or from Fukushima prefecture and delivered their babies in Fukushima prefecture on or after March 11, 2011 (excluding miscarriages, induced abortions, and stillbirths).	2011: 8575<br>2012: 6921<br>2013: 7022	Cross-sectional cohort	2011–2013	Free opinion	Ratio of opinion about radiation effect on fetus was decreasing overtime<br><br>Descriptive data:<br>2011:12.6%(29.8%), 2012: 5.6(26.8%); 2013: 2.0% (17.4%)<br>Ratio among response of free opinion are given in parentheses.
Intervention						modifiability
Hino et al. [18]	Explanatory meeting Participants (749 people in Fukushima and 50 outside Fukushima (in Yamagata) participated)	594	Pre-post no control arm	Sep-14 to Jun-15	Likert scale for anxiety regarding the effects of radiation on the thyroid (1–10)	Change anxiety from participation explanatory meeting<br><br>Moderator of the effect:<br>sex (woman > man),<br>size (49–99 > more than 100),<br>satisfaction with provided objective data and cancer information (not applicable > applicable)
Sugimoto et al. [20]	Residents in Soma city: Participants in a radiation-health-seminar for a total of 1560 residents, at 12 different locations in Fukushima	1560	Pre-post no control arm	Jun-11 to Jul-11	Radiation/health fear, fears for future. fears about social disruption	All three factors showed a statistically significant decrease from pre- to post- seminar testing (paired t-test p-value,0.0001 in all cases).

Midorikawa et al. [29]	Explanatory meetings for parents of tested children	501	Descriptive	Since 2013	Pre post meeting anxiety concerning effects of radiation on the thyroid (decrease, no change, and increase)	Decreased: 60.1%, unchanged: 30.7%; increased: 5.6%, unknown:3.6%
	Immediate post examination individual counseling	453	Descriptive	Oct-14	Assuaging of anxiety (5 point likert type scale	Relieved(5): 69%, relieved(4): 19%, (3): 8.8%, (2): 2.4%, (1): 0.9% not relieved
Fujii et al. [30]	Fifty-eight groups of parents and two expecting mothers participated in the one-to-one consultations with a radiologist held in Nagareyama city.	No detail available	Descriptive	No detail available	Anxiety and fear of the radiation exposure issues	
Imamura et al. [31]	Mothers living with a preschool children in Fukushima city or neighboring smaller municipalities	37 (intervention: 18, control: 19)	Randomized two arm controlled trial Permuted-block randomization waiting list	Aug-14 to Nov-14	This scale consists of seven items asking about the respondents' fears and worries of effects of radiation exposure on their own health and the next generation's health, and the effect of news reports on the accident at the nuclear power plant, among others. The response options (item scores) were on a four-point scale, from totally disagree (1) to totally agree (4). The total scale scores ranged from 7 to 28.	No statistical significant decrease was showed at post, 1 month, and 3month after the intervention
